# Predicting head and neck cancer response to radiotherapy with a chemokine-based model

**DOI:** 10.1038/s41598-025-13346-z

**Published:** 2025-08-04

**Authors:** Jinzhi Lai, Rongfu Huang, Jingshan Huang

**Affiliations:** 1 Department of Oncology, The Second Affiliated Hospital of Fujian Medical University, Quanzhou, 362000 Fujian China; 2 Department of Clinical Laboratory, The Second Affiliated Hospital of Fujian Medical University, Quanzhou, 362000 Fujian China; 3 Department of General Surgery, The Second Affiliated Hospital of Fujian Medical University, Quanzhou, 362000 Fujian China

**Keywords:** Genetics, Biomarkers

## Abstract

Radiotherapy resistance remains a major challenge in Head and neck squamous cell carcinoma (HNSCC) treatment. This study aimed to develop a chemokine-based model for predicting radiosensitivity in HNSCC using a retrospective analysis of 432 patients from the TCGA database. We identified a model incorporating CXCL2, CCL28, and CCR8 expression that effectively stratified patients into radiosensitive (RS) and radioresistant (RR) groups. Patients in the RS group demonstrated significantly improved overall survival (OS) with radiotherapy, whereas this prognostic advantage was not observed in the non-radiotherapy group. Notably, patients within the RS group with high PD-L1 expression exhibited even better OS and increased immune infiltration, indicating a synergistic relationship between radiosensitivity and PD-L1 expression. Further analyses revealed enrichment of immune-related pathways and higher effector immune cell abundance in the RS group, suggesting greater potential for immunotherapy response. Corroborating these findings, analysis of the GSE40020 cohort showed significant upregulation of CCL28 in patients with complete response compared to those with post-treatment failure. In vitro experiments using radiosensitive and radioresistant Tongue squamous cell carcinoma (TSCC) cell lines validated the association between chemokine gene expression and radiosensitivity. Our model provides a valuable tool for identifying HNSCC patients who may benefit from combined treatment strategies incorporating synergistic anti-tumor agents.

**Supplementary Information:**

The online version contains supplementary material available at 10.1038/s41598-025-13346-z.

## Introduction

Head and neck squamous cell carcinoma (HNSCC) is the sixth most common malignancy worldwide, with approximately 900,000 new cases and over 350,000 deaths reported annually^1^. The incidence and mortality rates of HNSCC are continually increasing, highlighting the urgency for effective treatment strategies^2^. Currently, the standard treatment for HNSCC involves concurrent chemoradiotherapy (CRT). For early-stage HNSCC, both surgery and radiotherapy yield similar outcomes, with a 5-year survival rate ranging from 70–90%^3^. However, despite the availability of various treatment modalities such as surgery, radiotherapy, chemotherapy, and targeted therapy, the 5-year survival rate for patients with locally advanced HNSCC undergoing CRT remains below 60%^4^. A significant challenge in the treatment of HNSCC is radio-resistance, which is a major cause of local tumor recurrence and radiotherapy failure^5^. Overcoming radio-resistance and enhancing radiosensitivity are critical objectives in HNSCC treatment. Therefore, there is an urgent need for novel therapeutic approaches that can effectively overcome radio-resistance and for strategies to identify and select patients who are more likely to respond favorably to radiotherapy in HNSCC.

Chemokines are a class of small soluble proteins that promote cell differentiation, migration, and transport^6^. They induce the directed migration of cells expressing chemokine receptors to specific tissues or organs. In the tumor microenvironment, chemokines play complex roles^7^. They not only participate in the recruitment and activation of immune cells but also influence tumor response to therapy. Recent research has revealed that changes in the expression of chemokines and their receptors are closely associated with the radiosensitivity of tumors^8^. Yang et al. demonstrated that targeting tumor-derived CXCL1 or blocking the CXCL1-CXCR2 signaling pathway could restore the radiosensitivity of radioresistant esophageal squamous cell carcinoma (ESCC) xenografts in vivo, indicating the potential of CXCL1 as a predictive biomarker for radiosensitivity in ESCC^9^. However, the specific roles of chemokines and their receptors in radiosensitivity, particularly in HNSCC, remain poorly understood. Therefore, investigating the intricate relationship between chemokines and the radiosensitivity of HNSCC is warranted.

The advent of precision medicine has catalyzed a remarkable integration of genomic and transcriptomic data, fundamentally transforming our comprehension of cancer heterogeneity^10^. Extensive research has utilized cellular and animal models to identify molecular signatures predictive of radiosensitivity in cancer^11–13^. For example, Torres-Roca et al. constructed a 10-gene radiosensitivity index that demonstrated robust predictive power in 48 cancer cell lines^14^. Furthermore, Kim et al. developed a 31-gene signature specifically associated with radiotherapy response^15^. Nevertheless, there remains a significant gap in our knowledge regarding the potential of chemokine-based models as biomarkers for predicting the radiotherapy response in HNSCC. A more thorough investigation of these chemokine signatures could not only elucidate the mechanisms underlying radio-resistance but also guide the selection of HNSCC patients who are most likely to benefit from radiotherapy.

This study aimed to assess the utility of a novel chemokine-based radiosensitivity model in predicting the response to radiotherapy in HNSCC patients. We constructed a unique signature by integrating genes related to chemokines and their receptors, subsequently evaluating its efficacy in distinguishing patients likely to benefit from radiotherapy. The model’s predictive efficacy was evaluated by categorizing HNSCC patients into radiosensitive (RS) and radioresistant (RR) groups using this radiosensitivity risk model. Significant differences were observed between the RS and RR groups in terms of therapeutic response to radiotherapy. These findings highlight the potential of this chemokine-based radiosensitivity model to provide valuable insights into the heterogeneity of radiosensitivity in HNSCC. Importantly, this signature may serve as a tool for guiding the selection of optimal treatment regimens, including identifying potential synergistic combinations of radiotherapy with anti-tumor drugs.

## Materials and methods

### Data acquisition

Gene expression profiles and corresponding clinical data for patients with HNSCC were obtained from two publicly accessible databases: The Cancer Genome Atlas (TCGA) (https://portal.gdc.cancer.gov/projects/TCGA-HNSCC) and the Gene Expression Omnibus (GEO) (https://www.ncbi.nlm.nih.gov/geo/query/acc.cgi?acc = GSE40020)^16,17^. Inclusion criteria were: (1) primary HNSCC tumors, (2) complete follow-up data exceeding 30 days, and (3) comprehensive radiotherapy information. A total of 432 TCGA-HNSCC patients meeting these criteria, with both RNA sequencing and clinical data, were included. Genomic alterations data for patients with HNSCC were downloaded from TCGA database.

### Construction of the chemokine-based radiosensitivity model

A total of sixty-six chemokines and chemokine receptors, identified from prior literature, were incorporated into the analysis^18^. Univariate Cox regression analysis was conducted to identify differentially expressed chemokine-related genes (DE-CRGs) significantly associated with overall survival (OS) in radiotherapy patients, but not in non-radiotherapy patients. Subsequently, multivariate Cox regression was employed to develop the model based on the identified DE-CRGs in radiotherapy patients. The radiosensitivity risk score was calculated using the formula:

$$Radiosensitivity\;Risk\;score=\sum\limits_{{i=1}}^{n} {Coe{f_i}} *Gen{e_i}$$

Using the median value of the risk score, radiotherapy patients were classified into two groups: the RS group and the RR group. Patients in the non-radiotherapy cohort were assigned a radiosensitivity risk score using the same formula. Patients in the RS group exhibited improved survival outcomes compared to those in the RR group after receiving radiotherapy. However, in the absence of radiotherapy, survival rates between the RS and RR groups were not significantly different. Importantly, the RS group demonstrated a statistically significant improvement in OS with radiotherapy compared to without. Conversely, no significant difference in OS was observed in the RR group, regardless of radiotherapy. These conditions highlight the effectiveness of the radiosensitivity model in identifying patients who benefit from radiotherapy.

### Immune cell infiltration, function and pathway enrichment analysis

To characterize the tumor microenvironment and immune cell infiltration within the RS and RR groups, we employed the ESTIMATE algorithm to generate immune, stromal, and overall ESTIMATE scores^19^. Single-sample Gene Set Enrichment Analysis (ssGSEA) was then conducted to assess the enrichment levels of 29 immune-related signatures, each representing diverse immune-related functions and pathways^20^. Additionally, the CIBERSORT algorithm was employed to determine the infiltration levels of 22 different immune cell subtypes within each sample^21^. The CIBERSORT output was normalized to ensure the sum of all immune cell type fractions equaled one.

To investigate the differential cellular pathways between the RS and RR groups, we performed Gene Set Variation Analysis (GSVA) for Kyoto Encyclopedia of Genes and Genomes (KEGG) pathway enrichment^22^. This approach pinpointed the most significantly enriched molecular pathways distinguishing the two groups. Additionally, Gene Ontology (GO) analysis was conducted to obtain a detailed understanding of the biological processes (BP), molecular functions (MF), and cellular components (CC) associated within the two groups.

### Estimating treatment response through simulated analysis

To evaluate the potential response of HNSCC patients to various therapies, we utilized several predictive algorithms and databases. Immunotherapeutic response was evaluated using the Tumor Immune Dysfunction and Exclusion (TIDE) score, encompassing T cell dysfunction and exclusion scores, retrieved from the TIDE portal (http://tide.dfci.harvard.edu/)^23^. Further informing immunotherapy response prediction, we obtained Immunophenotype Scores (IPS) specific to CTLA-4 and PD-1 blockade from The Cancer Immunome Atlas (TCIA) database (https://tcia.at/home)^24^. These scores, ranging from 0 to 10, are calculated based on the expression levels of representative gene sets and provide a nuanced assessment of immunotherapy efficacy. For predicting responses to chemotherapy and targeted therapies, we employed the pRRophetic R package^25^. This tool uses data from the Genomics of Drug Sensitivity in Cancer (GDSC) database to predict drug sensitivity by estimating the half-maximal inhibitory concentration (IC50) for various drugs.

### Cell culture and radioresistant HNSCC cell line development

The human tongue squamous cell carcinoma (TSCC) cell line, CAL-27, was obtained from the China Center for Type Culture Collection (CCTCC, Wuhan, China). Cells were cultured in RPMI 1640 medium (Corning, United States) supplemented with 10% fetal bovine serum (Corning, United States) and 1% antibiotics (Gibco-BRL, Gaithersburg, MD, United States). Cultures were maintained at 37 °C in a humidified atmosphere with 5% CO2. Mycoplasma contamination was ruled out prior to experimentation. To establish a radioresistant subline (CAL-27IR), CAL-27 cells were subjected to incremental radiation doses (2, 4, 6, 8, 10, 10, and 10 Gy) over seven cycles, culminating in a total dose of 50 Gy^26^. The radio-resistance of CAL-27IR was subsequently evaluated using cell proliferation assays. To evaluate the radio-resistance of CAL-27IR cells, a Cell Counting Kit-8 (CCK-8) assay was employed. Briefly, CAL-27IR and CAL-27 cells were seeded in 96-well plates and exposed to varying radiation doses. Following irradiation, cells were incubated with CCK-8 solution, and cell viability was determined by measuring the optical density (OD) at 450 nm using a microplate reader. The OD450 values were directly proportional to cell viability.

### Quantitative real-time polymerase chain reaction (qRT-PCR)

Total RNA was isolated from cells using TRIzol reagent (Invitrogen, San Diego, CA, USA) according to the manufacturer’s protocol. RNA concentration and purity were assessed using a NanoDrop 2000 spectrophotometer (Thermo Fisher Scientific, USA). To quantify mRNA expression levels of target genes, qRT-PCR was conducted using the SYBR PrimeScript RT-PCR Kit (Invitrogen, USA) following the provided protocol. Primer sequences for the target genes are listed in Supplementary Table S1. All qRT-PCR assays were performed in triplicate, including appropriate negative controls to ensure specificity. Threshold cycle (Ct) values for target genes were normalized against the geometric mean of Ct values for internal control genes, such as GAPDH. Relative gene expression levels were calculated using the 2^−ΔΔCt^ method and expressed as fold changes relative to the internal controls.

### Statistical analysis

Statistical analyses were conducted using R software version 4.1.3. The selection of statistical tests was determined by the data type and distribution. For comparisons involving categorical variables across different groups, the Chi-square test was used. To compare continuous variables between two independent groups, the Mann-Whitney U test was applied. For comparisons among multiple independent groups, the Kruskal-Wallis test was utilized. Pearson’s correlation coefficient evaluated linear relationships between normally distributed variables, while Spearman’s rank correlation coefficient was used for non-parametric data with non-normal distributions. Survival outcomes between groups were analyzed using Kaplan-Meier curves, with statistical significance assessed through the log-rank test. All statistical tests were two-tailed to detect both positive and negative associations. A significance level of *p* < 0.05 was applied throughout the study unless otherwise specified.

## Results

### Chemokine-based risk model for predicting radiotherapy response in HNSCC

**Fig. 1:**
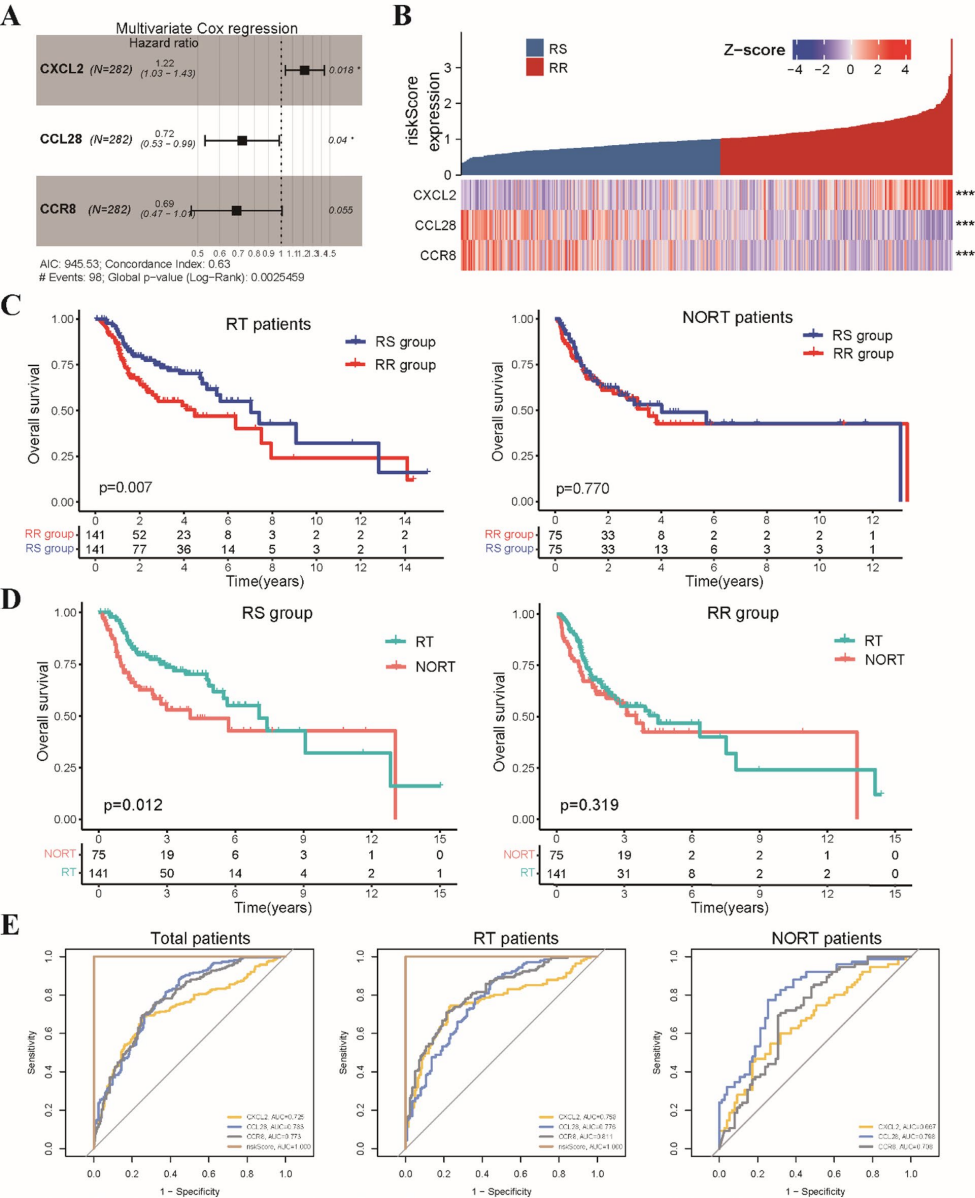
Construction of a chemokine-based radiosensitivity model in the TCGA-HNSCC cohort. **(A)** Forest plot illustrating hazard ratios and corresponding 95% confidence intervals for the three genes comprising the radiosensitivity model, as determined by multivariate Cox regression analysis. **(B)** The heatmap visualizes the expression profiles of the three genes composing the radiosensitivity model across RS and RR groups. **(C)** The Kaplan-Meier curves depicts the impact of radiosensitivity on OS stratified by radiotherapy status (RT/Non-RT patients). **(D)** The Kaplan-Meier curves illustrates OS within each radiosensitivity group (RS/RR groups) for patients receiving radiotherapy compared to non-radiotherapy. **(E)** ROC curves showing the capacity of risk model to predict radiosensitivity in the HNSCC patients. * *p* < 0.05, *** *p* < 0.001.

To comprehensively evaluate the potential of chemokine expression patterns in predicting radiotherapy response and guiding personalized treatment strategies, we developed a chemokine-based radiosensitivity prediction model (Fig. S1). Our study encompassed 47 chemokines and 19 chemokine receptors, analyzing their association with patient prognosis in both radiotherapy (RT) and non-radiotherapy (NORT) groups. Univariate Cox regression analysis revealed four chemokine and chemokine receptor genes significantly associated with OS exclusively in patients receiving radiotherapy (Fig. S2A-B). Finally, three of the four genes formed the foundation for a multivariate Cox regression model, generating a quantitative risk score for individual patients (Fig. 1A). The risk score was calculated as follows: Risk score = (expression of CXCL2 × 0.19507) + (expression of CCL28 × -0.32355) + (expression of CCR8 × -0.3691).

Patients receiving radiotherapy were stratified into RR (high-risk) and RS (low-risk) groups based on the median risk score. Patients in the non-radiotherapy group were also stratified into RS and RR groups using the same risk score formula developed for the radiotherapy group (Fig. 1B). Kaplan-Meier survival analysis demonstrated a significant OS benefit for patients in the RS group compared to the RR group within the radiotherapy group. Notably, this prognostic distinction was absent in the non-radiotherapy group, underscoring the model’s specificity to radiotherapy response (Fig. 1C). Furthermore, we employed a matched-pair analysis to directly compare OS between radiotherapy and non-radiotherapy patients within each risk group. The RS group exhibited significantly improved OS with radiotherapy compared to without, while no significant difference was observed in the RR group (Fig. 1D). In addition, the RS group also demonstrated superior disease-specific survival (DSS) compared to other groups (Fig. S2C). Receiver-operating characteristic (ROC) curve analysis with area under the curve calculation further validated the model’s accuracy in predicting radiosensitivity in HNSCC patients (Fig. 1E). Collectively, our findings suggest that this chemokine-based radiosensitivity prediction model represents a promising radiosensitivity signature for personalizing radiotherapy treatment in HNSCC.

### Correlation of radiosensitivity model with clinical characteristics and functional annotation

**Fig. 2:**
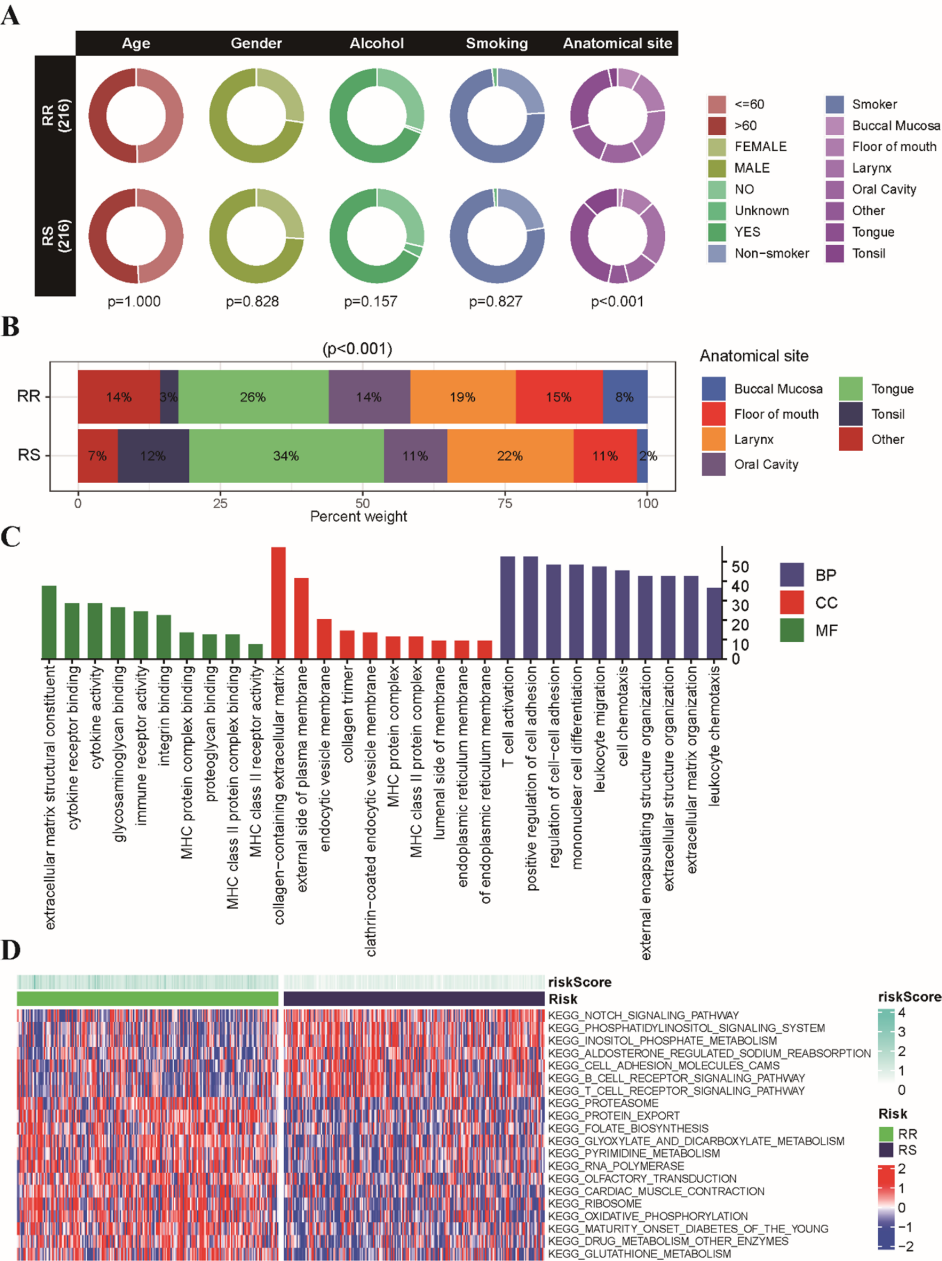
Association of the radiosensitivity model with clinicopathological features and functional annotations in HNSCC patients. **(A)** Circos plot illustrating the distribution of age, gender, smoking status, drinking status, and anatomical site between the RS and RR groups. **(B)** Stacked bar chart depicting the proportion of patients with different anatomical sites between these two groups. **(C)** Plot showcasing the top 10 enriched GO terms associated with DEGs in the BP, CC and MF categories. **(D)** Heatmap visualizing the enrichment scores of the top differentially enriched hallmark pathways between the RS and RR groups.

To explore potential associations between the radiosensitivity model and clinicopathological features, we conducted a comparative analysis of patient characteristics within the RS and RR groups. The radiation-related data are summarized in Table S2, which includes total radiation doses and fractionated radiation dose for both groups. No statistically significant differences were observed between the two groups regarding T stage, N stage, M stage, pathological grade, age, gender, smoking status, or drinking status (Fig. S3A and Fig. 2A). However, analysis of anatomical site revealed a distinct pattern: the RS group exhibited a significantly higher prevalence of tumors originating in the tongue and tonsil, whereas tumors of the floor of mouth and buccal mucosa were more common in the RR group (Fig. 2B).

We further conducted a comprehensive analysis of gene expression profiles between the RS and RR groups. A total of 471 DEGs exhibited significant variations between the two groups (Fig. S3B). The GO enrichment analysis revealed that these DEGs were prominently associated with membrane transporter activity, immune cell chemotaxis, and migration, suggesting a potential role for immune-related processes in radiosensitivity (Fig. 2C). The results of GSVA enrichment analysis indicated that the RS group exhibited significant enrichment in pathways related to Notch signaling, T cell receptor signaling, and B cell receptor signaling, all of which are crucial for immune regulation and response (Fig. 2D). These findings indicate that the divergent responses to radiotherapy observed between the RS and RR groups may be intricately linked to variations in immune-related pathways and cellular processes.

### Exploring the connection between the radiosensitivity model and genomic characteristics

**Fig. 3:**
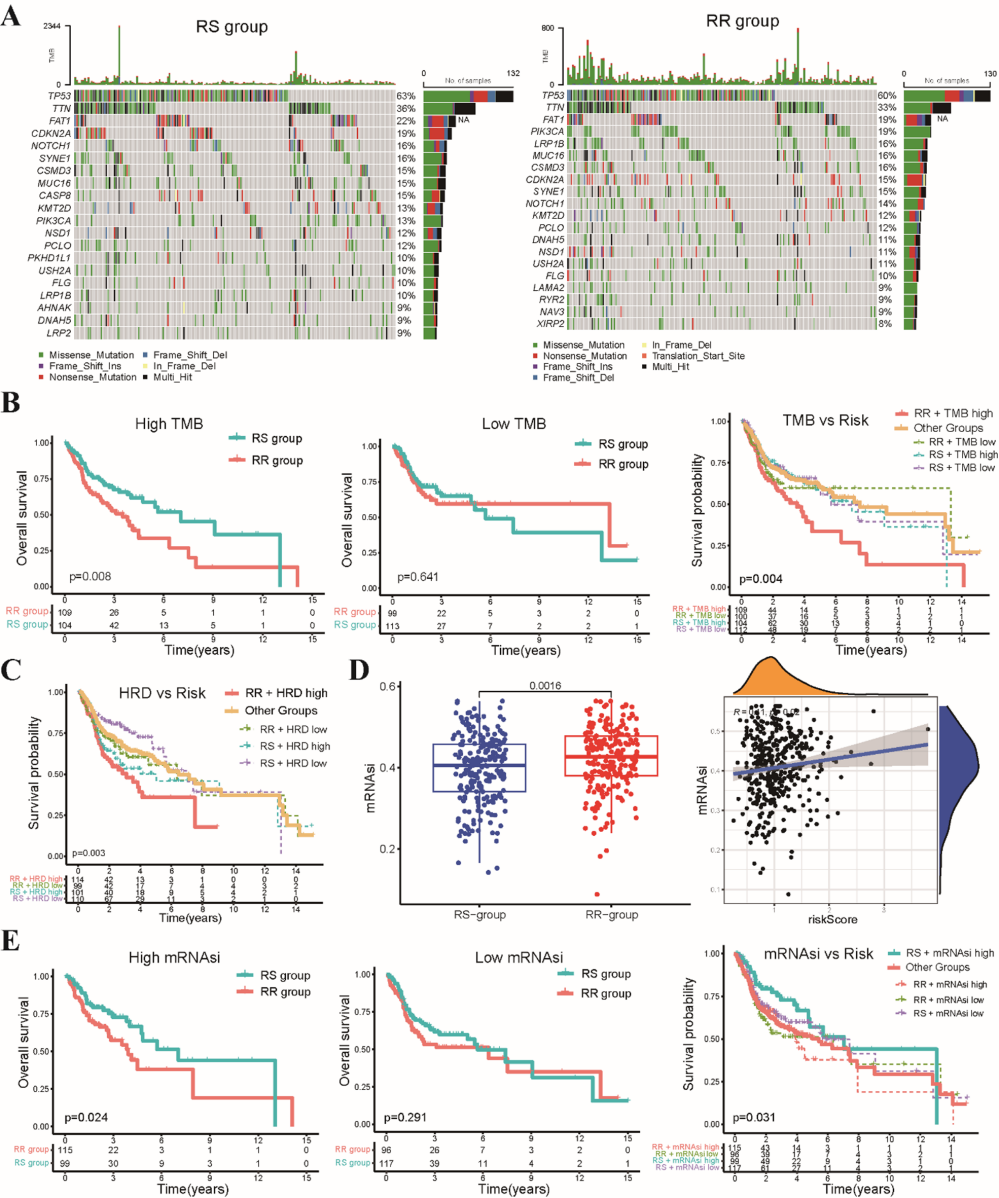
Interplay between radiosensitivity model and genomic characteristics in HNSCC patients. **(A)** Waterfall plots illustrating the top 20 most frequently mutated genes within both the RS and RR groups. **(B)** Kaplan-Meier curve showcasing the OS disparity between patients in the RR group with high TMB values compared to all other patient groups. **(C)** Kaplan-Meier survival curves depicting OS differences between patients in the RR group with high HRD scores and all other patient subgroups. **(D)** Scatter plot visualizing the distribution of mRNAsi scores across the RS and RR groups. **(E)** Kaplan-Meier survival curves demonstrating OS comparisons between patients in the RS group displaying high mRNAsi scores and all other patient subgroups.

The influence of tumor mutational burden (TMB) on cancer prognosis remains a topic of debate. To shed light on the relationship between genomic features and radiosensitivity, we analyzed data from patients with available mutation and copy number variation (CNA) information. The top 5 most frequent mutations were consistent between the RS and RR groups: TP53, TTN, FAT1, PIK3CA, and CDKN2A (Fig. 3A). While we did not find a direct association between our radiosensitivity model and TMB scores when comparing the RS and RR groups (Fig. S4A), subgroup analyses revealed a crucial distinction. Patients in the RR group with high TMB values exhibited significantly worse OS compared to the RS group, whereas no significant difference in OS was observed between the groups for patients with low TMB values, indicating high TMB specifically within the RR group is associated with a poorer prognosis (Fig. 3B). Similarly, subgroup analysis of homologous recombination deficiency (HRD) scores mirrored the TMB findings: high HRD within the RR group correlated with a worse prognosis compared to other subgroups (Fig. 3C). Given the emerging recognition of cancer stemness as a key factor in radiotherapy response, we further investigated the relationship between our risk model and mRNAsi scores. We observed that mRNAsi scores were significantly lower in the RS group compared to the RR group (Fig. 3D). Subgroup analyses demonstrated that patients in the RS group with high mRNAsi values had significantly better OS compared to the RR group, while no significant difference in OS was found between the groups for patients with low mRNAsi values. This indicates that low mRNAsi within the RS group is associated with a more favorable clinical outcome (Fig. 3E).

### Association of radiosensitivity model with tumor immune microenvironment

**Fig. 4:**
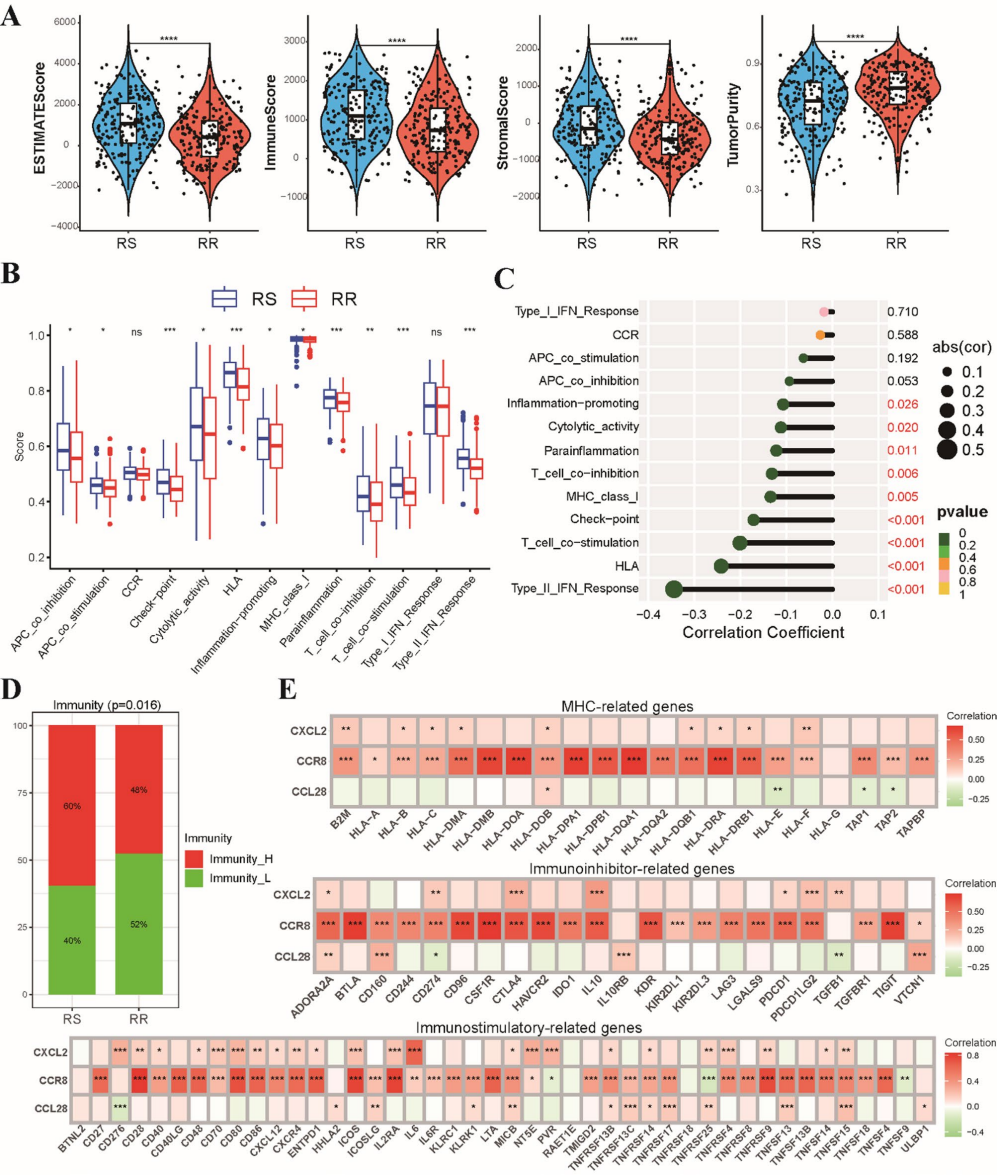
Characterization of the tumor immune microenvironment in RS and RR groups based on ESTIMATE algorithm. **(A)** Violin plots illustrating the distribution of ESTIMATE scores, stromal scores, immune scores, and tumor purity, between the RS and RR groups. **(B)** Bar chart depicting the enrichment scores of immune-related functions and pathways in the RS and RR groups. **(C)** Lollipop plot showcasing the correlation between three genes comprising the radiosensitivity model and the enrichment scores of immune-related functions and pathways. **(D)** Stacked histogram visualizing the proportions of patients classified as high-immunity or low-immunity within the RS and RR groups. **(E)** Correlation analysis demonstrating the relationships between three radiosensitivity model genes and the expression levels of immunoinhibitor-related, immunostimulatory-related, and MHC-related genes. * *p* < 0.05, ** *p* < 0.01, *** *p* < 0.001.

Mounting evidence points towards the tumor immune microenvironment playing a crucial role in dictating tumor response to radiotherapy. To delve into this intricate relationship, we first characterized the immune landscape of tumors using the ESTIMATE algorithm. Our analysis revealed that RS group exhibited significantly higher ESTIMATE, immune, and stromal scores. Conversely, the RR group displayed higher tumor purity, suggesting a lower infiltration of immune and stromal components (Fig. 4A). To further dissect the immune landscape, we employed ssGSEA on the transcriptomes, evaluating the enrichment of 29 immune-related cell types, functions, and pathways. This analysis revealed a widespread upregulation of immune-related functions and pathways within the RS group compared to the RR group (Fig. 4B). Notably, correlation analysis highlighted a strong association between the RS group and key immune features, including T cell co-stimulation/co-inhibition, interferon (IFN) response, and MHC molecule presentation (Fig. 4C). Clustering analysis based on ssGSEA scores allowed us to categorize HNSCC patients into high-immunity and low-immunity clusters (Fig. S4B). Interestingly, the RS group was predominantly composed of patients belonging to the high-immunity cluster (Fig. 4D). Finally, we investigated the interplay between our three model genes and various immune-related genes curated from the TISIDB database, including immunoinhibitor-related, immunostimulatory-related and MHC-related genes. Our analysis revealed CCR8 exhibited positive correlations with numerous immune-related genes, while CCL28 showed positive correlations specifically with immunoinhibitory and MHC-related genes (Fig. 4E). These findings underscore the intricate connection between our radiosensitivity model and the tumor immune microenvironment in HNSCC.

### Linking the radiosensitivity model to tumor-infiltrating immune cells

**Fig. 5:**
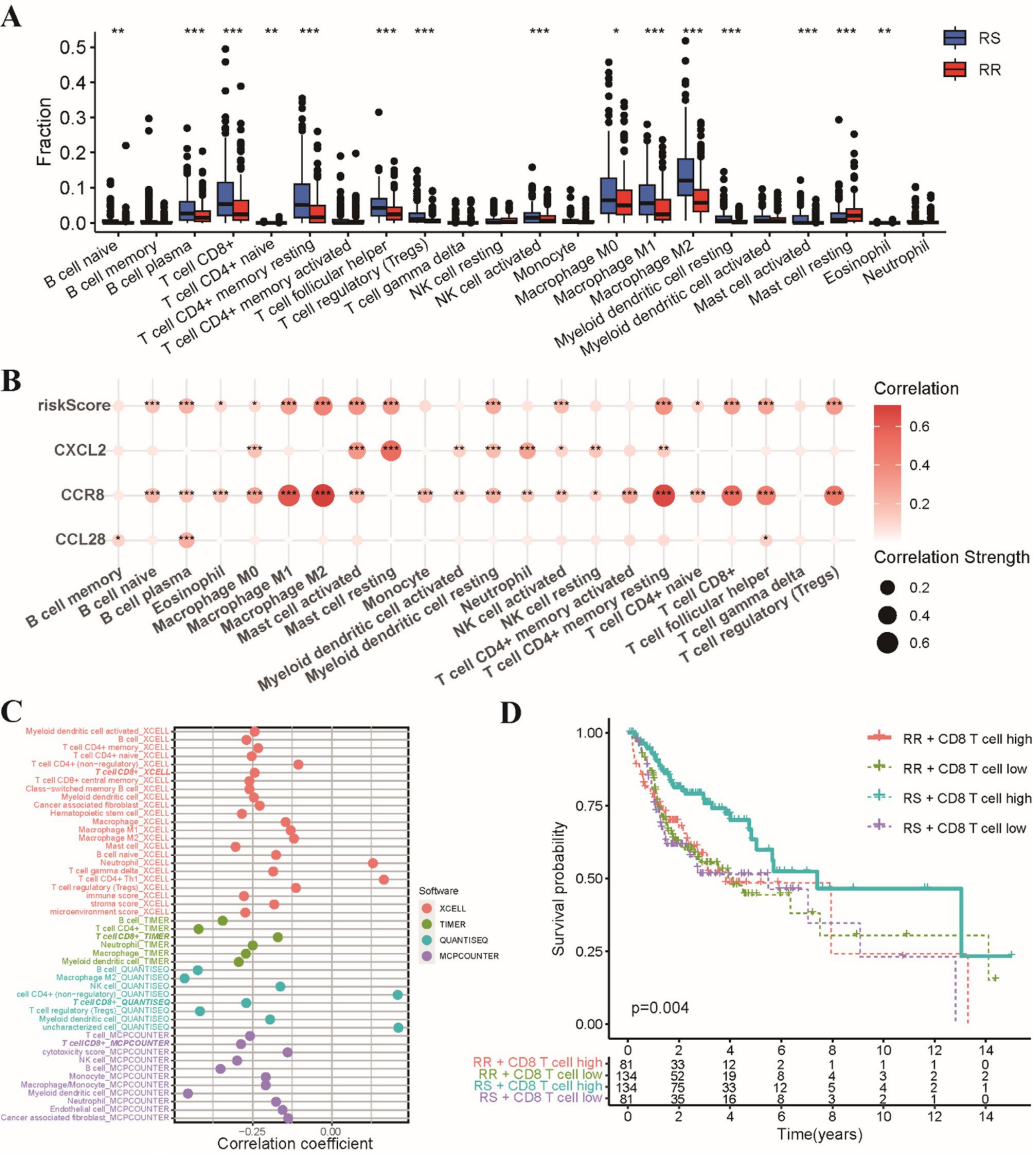
Immune cell infiltration patterns between RS and RR groups based on CIBERSORT algorithm. **(A)** Bar chart illustrating the relative abundance of 22 distinct immune cell types within the RS and RR groups, as determined by CIBERSORT analysis. **(B)** Bubble map visualizing the pearson correlation coefficients between the expression levels of the three genes comprising the radiosensitivity model and the infiltration levels of 22 immune cell types. **(C)** Comparative analysis of CD8 T cell infiltration levels across the RS and RR groups, as assessed by four independent algorithms: XCELL, TIMER, QUANTISEQ, and MCPCOUNTER. **(D)** Kaplan-Meier survival curves demonstrating the prognostic significance of CD8 T cell infiltration in radiotherapy patients. *p < 0.05, **p < 0.01, ***p < 0.001.

To further elucidate the intricate relationship between our radiosensitivity model and the immune cell landscape, we employed the CIBERSORT algorithm to deconvolute the immune cell composition. Our analysis unveiled the RS group exhibited a significantly higher abundance of CD8 T cells, regulatory T cells (Tregs), and M1/M2 macrophages. In contrast, the RR group displayed a higher prevalence of resting mast cells and eosinophils (Fig. 5A). We performed correlation analyses to investigate the associations between the expression levels of our three radiosensitivity genes and the infiltration levels of various immune cell populations. Notably, most immune cells exhibited positive correlations with our risk model (Fig. 5B). This observation is particularly noteworthy as CD8 T cells are key players in orchestrating antitumor immune responses, including responses to radiotherapy. To validate these findings, we leveraged multiple independent algorithms, namely TIMER, XCELL, MCPCOUNTER, and QUANTISEQ, to assess CD8 T cell infiltration. Consistent with our initial observations, all four algorithms confirmed that the RS group displayed a significantly higher abundance of CD8 T cells compared to the RR group (Fig. 5C). Furthermore, subgroup analyses revealed that patients within the RS group who exhibited high CD8 T cell infiltration experienced superior clinical outcomes compared to other patient subgroups (Fig. 5D). This finding underscores the potential of CD8 T cells as a critical determinant of response to radiotherapy in the context of our radiosensitivity model.

### Exploring the antitumor efficacy potential of the chemokine-based radiosensitivity model

**Fig. 6:**
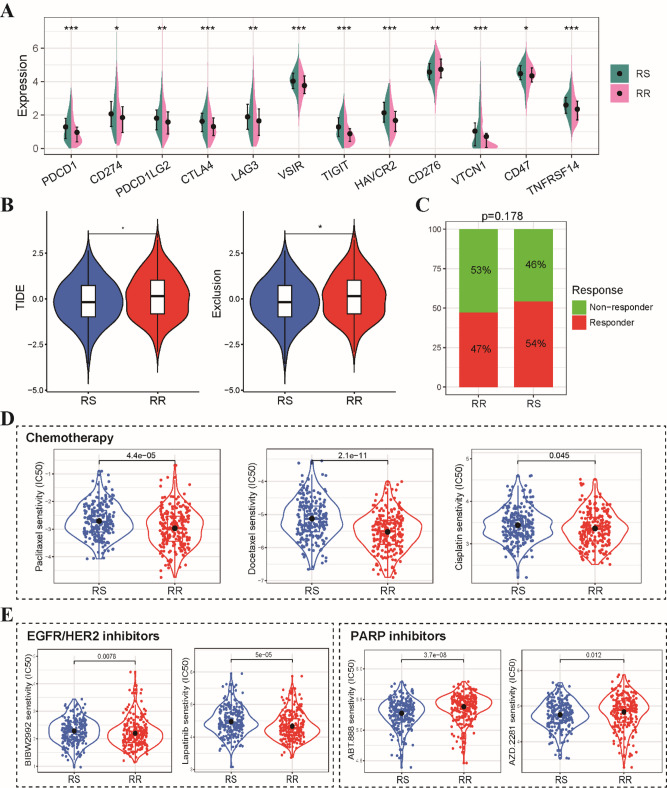
Predicted differential sensitivity to immunotherapy, chemotherapy, and targeted therapy based on TIDE and pRRophetic algorithms. **(A)** Comparative analysis of the expression levels of key immune checkpoint genes between the RS and RR groups. **(B)** Violin plots illustrating the distribution of TIDE scores and T cell exclusion scores within the RS and RR groups. **(C)** Stacked bar chart depicting the proportion of patients classified as responders or non-responders to immunotherapy within each risk group. **(D)** Boxplots showcasing the IC50 values for conventional first-line chemotherapeutic agents (paclitaxel, docetaxel, and cisplatin) in the RS and RR groups. **(E)** Boxplots comparing the IC50 values for EGFR/HER2 inhibitors and PARP kinase inhibitors between the RS and RR groups. *p < 0.05, **p < 0.01, ***p < 0.001.

With immunotherapy emerging as a revolutionary force in cancer treatment, we sought to explore the immunotherapeutic implications of our chemokine-based radiosensitivity model in HNSCC. We began by comparing the expression levels of various immune checkpoint markers between the RS and RR groups. As anticipated, the RS group exhibited significantly higher expression levels of most immune checkpoint genes, with the exception of CD276 (Fig. 6A). Subsequently, we employed the TIDE algorithm to simulate and predict immunotherapy responses based on gene expression profiles. Our analysis revealed that the RS group had lower TIDE scores and exclusion scores compared to the RR group. These findings collectively indicate a higher likelihood of favorable immunotherapy responses in the RS group (Fig. 6B). Although the proportion of patients responding to immunotherapy was higher in the RS group compared to the RR group, the difference did not reach statistical significance (Fig. 6C). Considering the importance of chemotherapy in HNSCC treatment, we utilized the pRRophetic R package to simulate and predict drug sensitivity patterns for chemotherapeutic agents based on gene expression profiles. Our analysis showed that the RS group had higher IC50 values for several first-line chemotherapeutic agents, including paclitaxel, docetaxel, and cisplatin, compared to the RR group. This suggests that patients in the RR group may be more sensitive to these conventional chemotherapy regimens (Fig. 6D). Interestingly, we observed distinct patterns of sensitivity for targeted therapies. The RS group displayed higher IC50 values for EGFR/HER2 inhibitors, indicating potential resistance to these agents. Conversely, the RS group exhibited lower IC50 values for PARP inhibitors, suggesting increased sensitivity to this class of drugs (Fig. 6E). These findings highlight the potential of integrating our radiosensitivity model with chemotherapy and immunotherapy response predictions to develop personalized treatment strategies for HNSCC patients.

### Integrating the radiosensitivity model and PD-L1 status for prognosis and treatment stratification

**Fig. 7:**
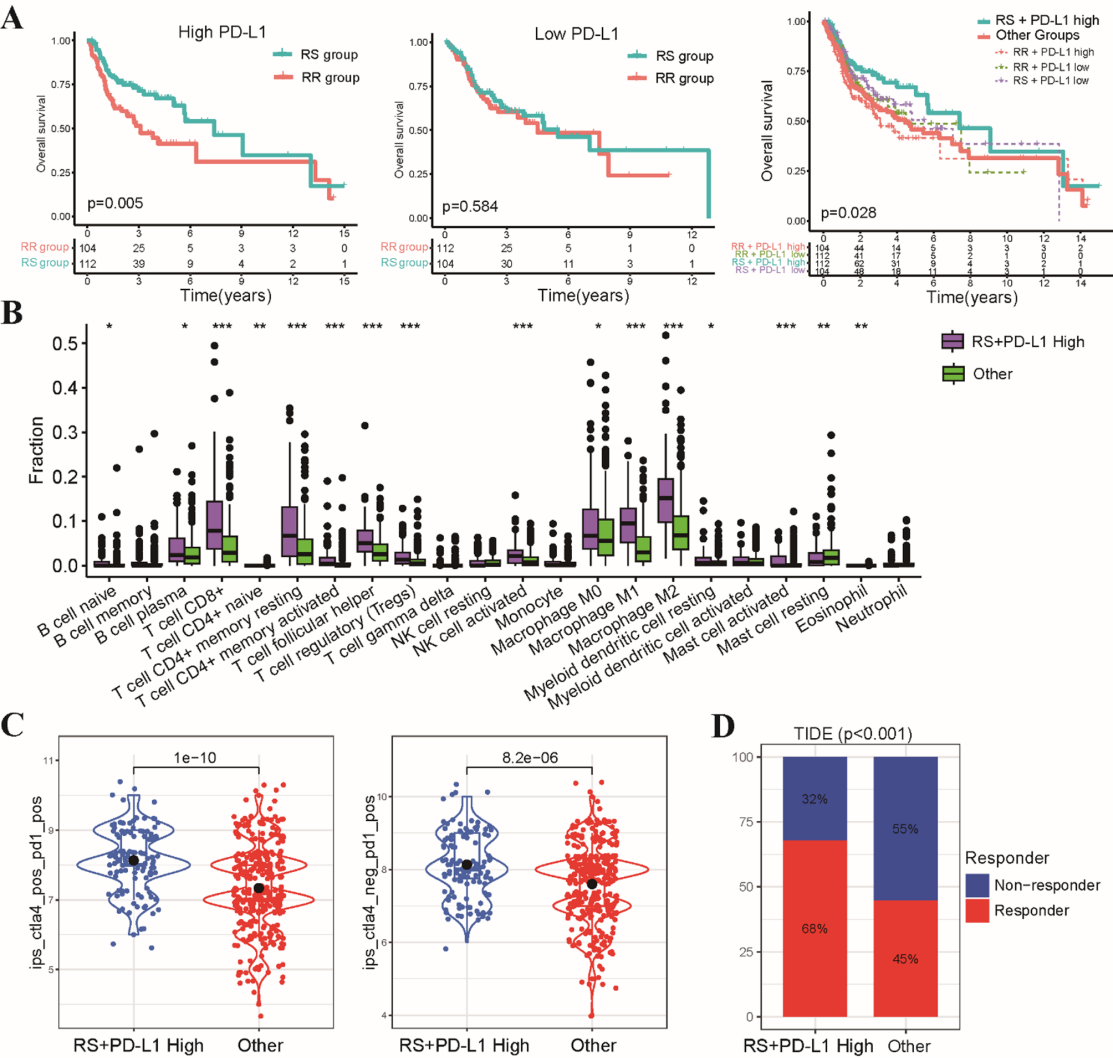
Integration of radiosensitivity and PD-L1 status for enhanced prognostication in HNSCC. **(A)** Kaplan-Meier survival curves depicting differences in OS between the RS-PD-L1-high subgroup and all other patient subgroups. **(B)** Comparative analysis of the proportions of 22 distinct immune cell infiltrates between the RS-PD-L1-high subgroup and all other subgroups. **(C)** Violin plots illustrating the distribution of IPS scores, predicting response to CTLA-4 and PD-1 inhibitors. **(D)** Stacked bar chart visualizing the proportion of patients classified as responders or non-responders to immunotherapy within the RS-PD-L1-high subgroup and other subgroups. *p < 0.05, **p < 0.01, ***p < 0.001.

To refine our understanding of the interplay between radiosensitivity and immunotherapy response, we investigated the prognostic value of PD-L1 expression within the framework of our radiosensitivity model. There was no difference in OS was observed between patients with high and low PD-L1 expression alone, or between those receiving radiotherapy or not (Fig. S5A). Interestingly, patients in the RS group with high PD-L1 expression exhibited significantly better OS compared to the RR group, whereas no significant difference in OS was observed between the groups for patients with low PD-L1 expression, indicating high PD-L1 expression within the RS group (RS-PD-L1-high subgroup) is associated with a better prognosis (Fig. 7A). The ESTIMATE analysis revealed revealed significantly higher stromal, immune, and composite ESTIMATE scores within the RS-PD-L1-high subgroup (Fig. S5B). Further supporting this result, CIBERSORT analysis demonstrated increased infiltration of various immune cell types, including CD8 T cells, CD4 T cells and follicular helper T cells, within the RS-PD-L1-high subgroup (Fig. 7B). Recognizing the important role of PD-L1 as a predictive biomarker for immunotherapy response, we sought to investigate its interplay with our radiosensitivity model. Employing the IPS algorithm, we observed a higher predicted response to PD-1 inhibitors within the RS-PD-L1-high group, suggesting a synergistic relationship between radiosensitivity and PD-L1 expression (Fig. 7C). Further corroborating this finding, the TIDE algorithm indicated a better response within the RS-PD-L1-high group (Fig. 7D and Fig. S5C). These findings collectively highlight the intricate interplay between PD-L1 expression and radiosensitivity in shaping immunotherapeutic response.

### In vitro validation of the chemokine-based radiosensitivity model

**Fig. 8:**
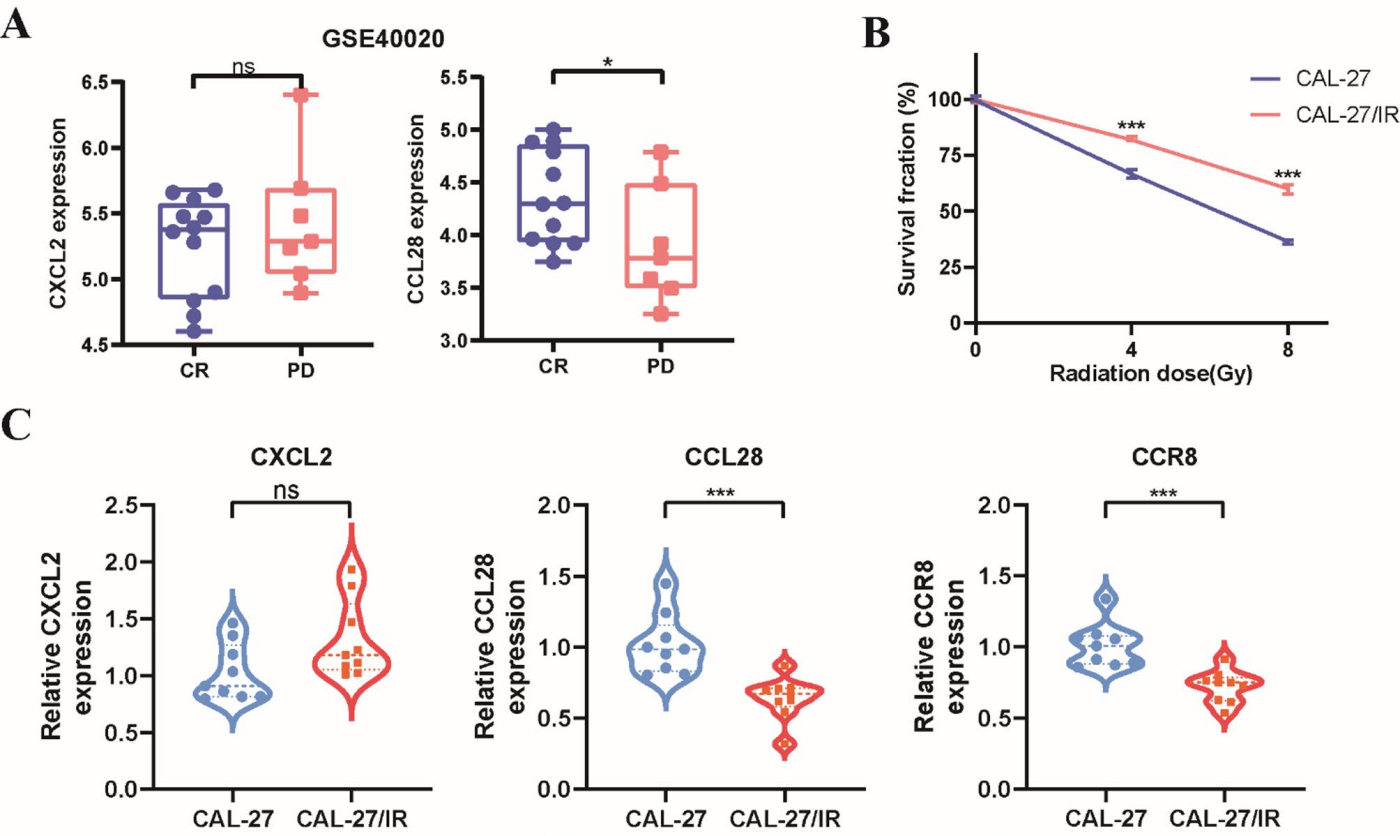
In vitro validation of the chemokine-based radiosensitivity model. **(A)** Differential expression of CXCL2, and CCL28 between complete response and post-treatment failure patients in the GSE40020 cohort. **(B)** Cell viability of CAL-27 and CAL-27/IR cells after exposure to varying doses of radiation, as assessed by CCK8 assay. **(C)** Expression levels of CXCL2, CCL28, and CCR8 in CAL-27 and CAL-27IR cells 24 h after in vitro irradiation. * *p* < 0.05, *** *p* < 0.001.

To validate the reliability of the chemokine-based radiosensitivity signature identified in our study, we validated this in independent dataset and in vitro cell lines. Analysis of the GSE40020 cohort from the GEO database revealed a significant upregulation of CCL28 expression in patients exhibiting complete response compared to those patients of post-treatment failure, while CXCL2 expression remained unchanged between these two groups (Fig. 8A). To further investigate these findings at the cellular level, we employed non-radioresistant (CAL-27) and radioresistant (CAL-27IR) cell lines. Cell viability was assessed using the CCK-8 assay following exposure to radiation. As expected, CAL-27IR cells exhibited significantly increased viability compared to CAL-27 cells after receiving 4 Gy and 8 Gy of radiation, confirming their acquired radio-resistance (Fig. 8B). Consistent with our bioinformatics analysis, we observed higher expression levels of CCL28 and CCR8 in the non-resistant CAL-27 cells compared to the resistant CAL-27IR cells. Interestingly, CXCL2 expression in CAL-27/IR cells showed an increasing trend after irradiation compared to non-irradiated CAL-27 cells, although this difference did not reach statistical significance (Fig. 8C).

## Discussion

Radiotherapy remains a cornerstone in the treatment of HNSCC. While modern imaging and radiotherapy techniques have significantly improved the precision of tumor localization, variability in patient response to radiotherapy persists^27^. Akervall et al. demonstrated that radiotherapy response in HNSCC patients is independent of TNM staging, with radio-insensitive or radio-resistant patients exhibiting a lower cause-specific survival^28^. Increasing evidence suggests that both inherent and adaptive radiation resistance are intricately linked to various biological functions^29^. Despite advancements in adapting radiotherapy fields to the anatomical specifics of tumors, the ability to tailor radiotherapy based on tumor biology remains a challenge due to the heterogeneity in individual responses. Our study aimed to develop a chemokine-based radiosensitivity prediction model for HNSCC and explore its potential implications for personalized treatment strategies. Our findings underscore the pivotal role of chemokines in modulating radiosensitivity, highlighting the potential of CXCL2, CCL28, and CCR8 as predictive biomarkers for radiotherapy response in HNSCC patients. The chemokine-based model effectively stratified patients receiving radiotherapy into RR and RS groups with significantly different prognosis. This stratification underscores the impact of intrinsic radiation resistance mechanisms on patient outcomes and highlights the potential for minimizing treatment-related toxicity through more tailored therapeutic approaches. The ability to accurately predict a patient’s response to radiotherapy is essential for optimizing treatment regimens, ultimately improving clinical outcomes and quality of life for HNSCC patients.

Deciphering the molecular basis of radiosensitivity is crucial for personalizing radiotherapy, as patients exhibiting inherent radio-resistance may necessitate more aggressive treatment strategies or alternative therapeutic modalities. High-throughput sequencing technology has emerged as a potent tool for elucidating the intricacies of individual radiosensitivity^30^. While the term “radiosensitivity” can have varying definitions depending on the context, in oncology settings, it is typically defined by two key criteria: (1) the demonstration of significantly enhanced survival benefits for the RS group compared to the RR group when both receive radiotherapy; and (2) the absence of a survival advantage for the RS group compared to the RR group in the absence of radiotherapy^31^. This clinically grounded definition provides a robust framework for evaluating our model. Despite extensive research on radiotherapy biomarkers, few have been rigorously validated in large cohorts, particularly by comparing their performance in radiotherapy versus non-radiotherapy groups^32^. This comparative analysis is crucial to differentiate predictive markers, which identify individuals likely to benefit from radiotherapy. Our chemokine-based model successfully captures both clinically relevant aspects of radiosensitivity, demonstrating its potential for accurately identifying patients who are likely to have a favorable response to radiotherapy.

In recent literature, the interplay between chemokines and the tumor microenvironment in HNSCC highlights their potential roles as biomarkers for therapeutic response. For instance, the CCR7/DUSP1 signaling axis has been shown to regulate the growth of HNSCC by modulating TGF-β1 secretion in inflammatory cancer-associated fibroblasts, highlighting a critical pathway that could influence therapeutic outcomes in HNSC^33^. Moreover, a strong correlation between CCL28 and CCR10 expression in oral squamous cell carcinoma (OSCC) emphasizes the importance of these chemokines in influencing tumor biology^34^. Lastly, the inhibition of CXCR1/2 has been reported to sensitize cancer cells to docetaxel, while also modifying the immune microenvironment in HPV-negative HNSCC, thus promoting anti-tumor immunity^35^. Collectively, these insights indicate that chemokine signaling pathways are not only vital for understanding tumor progression but also present promising targets for enhancing therapeutic efficacy in HNSCC.

The relationship between chemokines and response to radiotherapy is a complex and evolving area of research. Chemokines play a pivotal role in modulating the tumor microenvironment and influencing the effectiveness of radiotherapy^36^. One of the key mechanisms by which chemokines impact radiotherapy response is through the recruitment of immune cells to the tumor microenvironment^8^. The immune context, characterized by the balance of pro- and anti-tumor immune cells, significantly influences tumor response to radiation therapy. This recruitment can inhibit or activate anti-tumor immune responses, thereby shaping the tumor immune microenvironment and regulating the overall effectiveness of radiotherapy^37^. For example, Chen et al. reported that patients who responded well to radiotherapy had a higher aggregation of CD8 T cells post-treatment. This finding suggests that increasing CD8 T cell infiltration after radiotherapy could be an effective strategy to enhance tumor radiosensitivity^38^. Our study uncovered a compelling connection between chemokines and the tumor immune microenvironment. We found that the RS group exhibited a significantly higher abundance of CD8 T cells, Tregs, and M1/M2 macrophages. The observed enrichment of immune-related pathways in the RS group further supports this notion. These findings align with the growing body of evidence highlighting the crucial role of anti-tumor immunity in mediating radiotherapy efficacy. Our findings indicate that alterations in immune cell populations and their infiltration could significantly contribute to the emergence of tumor resistance to radiation. By understanding the intricate relationship between chemokines and the immune microenvironment, we can better appreciate the factors that dictate radiosensitivity.

One of the most noteworthy discoveries in this study is the potential predictive value of our risk model in forecasting anti-tumor responses in HNSCC. While immunotherapy has revolutionized cancer treatment, leading to improved clinical responses and overall survival, a substantial portion of HNSCC patients fail to respond to immune checkpoint inhibitors^39,40^. Our data indicate that the RS group exhibits a distinct immune landscape characterized by higher expression of immune checkpoints, including PD-1, PD-L1, CTLA-4, TIGIT, and TIM-3. This elevated expression profile suggests a more immunologically active microenvironment with a greater potential for anti-tumor responses. This observation offers a possible explanation for the observed higher likelihood of favorable immunotherapy responses in the RS group. Furthermore, we identified improved OS rates in the RS-PD-L1-high group compared to other cohorts. The superior survival in this group may be attributed to the presence of abundant immune cell infiltrates, which could contribute to heightened tumor sensitivity to radiation. This suggests a potential synergistic effect between radiotherapy and a pre-existing, robust anti-tumor immune response. Interestingly, while PD-L1 expression is often considered a mechanism for immune evasion, our findings suggest that in the context of a radiosensitive tumor, a high PD-L1 status may paradoxically be a positive prognostic factor. We hypothesize that the RS-PD-L1-high group may possess inherently more immunogenic tumors, characterized by a strong adaptive immune response, including a significant presence of tumor-infiltrating lymphocytes, before treatment initiation. This pre-existing immunological landscape, coupled with radiotherapy-induced recruitment of tumor-infiltrating lymphocytes, could contribute to enhanced tumor cell eradication and ultimately translate to improved long-term survival.

While predictive biomarkers for targeted therapies have significantly advanced clinical outcomes in certain cancer types, these effects are limited to a subset of patients^41,42^. Given the widespread use of radiotherapy across various cancer types, the radiosensitivity biomarkers have the potential to enhance treatment outcomes for a broad patient population^43^. Although our study identifies promising biomarkers for predicting radiosensitivity, the current evidence is insufficient to warrant their integration into routine clinical practice. However, several limitations need to be addressed. First, our analysis is based on retrospective datasets obtained from public databases. This necessitates prospective validation in larger, independent cohorts to ensure the robustness and applicability of our findings. Second, the intrinsic complexity and heterogeneity of HNSCC pose significant challenges. Variables such as HPV status, tumor subsite diversity, potentially affecting the reproducibility of our results. Third, while our study identifies CXCL2, CCL28, and CCR8 as relevant to radiosensitivity, their exact functional roles within this context require further experimental validation. Additionally, the TCGA database provides limited details on the specific doses (curative vs. palliative) and types of radiation therapy administered, which constrains our ability to fully characterize the patient population who received radiation therapy.

## Conclusions

In summary, our study identifies a novel chemokine-based radiosensitivity model with the potential to predict radiosensitivity in HNSCC. This model effectively stratifies patients into RS and RR groups, demonstrating strong associations with prognosis. Notably, our model’s association with genomic features, tumor-infiltrating immune cells and potential response to immunotherapy and chemotherapy underscores its potential for guiding treatment decisions and improving patient outcomes.

## Supplementary Information

Below is the link to the electronic supplementary material.

Supplementary Material 1

## Data Availability

The datasets generated or analyzed during the current study are available in the TCGA database (https://portal.gdc.cancer.gov/projects/TCGA-HNSCC) and Gene Expression Omnibus (https://www.ncbi.nlm.nih.gov/geo/query/acc.cgi?acc=GSE40020) database.
