# Supplementary material for: Predicting head and neck cancer response to radiotherapy with a chemokine-based model
Source: Sci Rep. 2025 Aug 4;15:28450. doi: 10.1038/s41598-025-13346-z (PMC12322276; doi:10.1038/s41598-025-13346-z)
Supplement: Supplementary file 1 — Supplementary Material 1 [file 41598_2025_13346_MOESM1_ESM.docx]

**Supplement Figures**

**
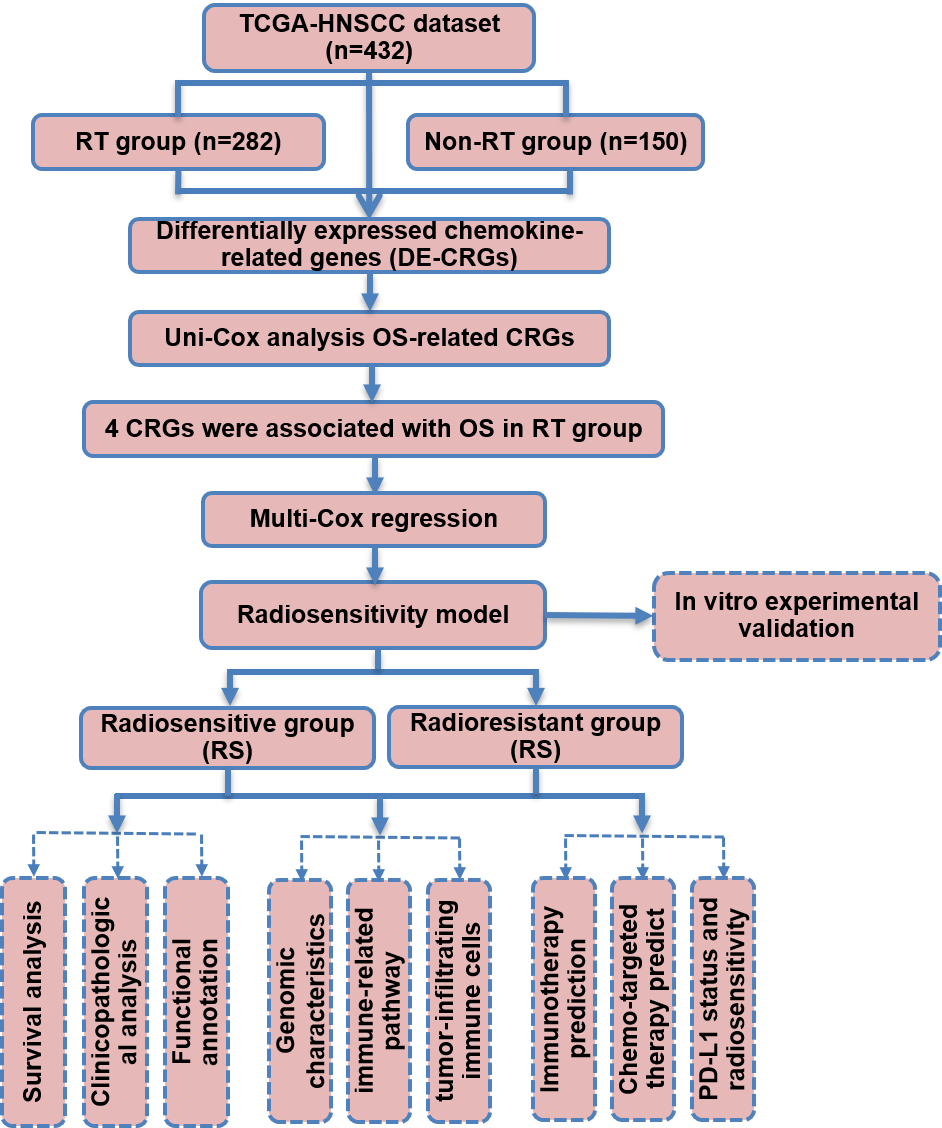
**

**Figure S1** The workflow of our study.


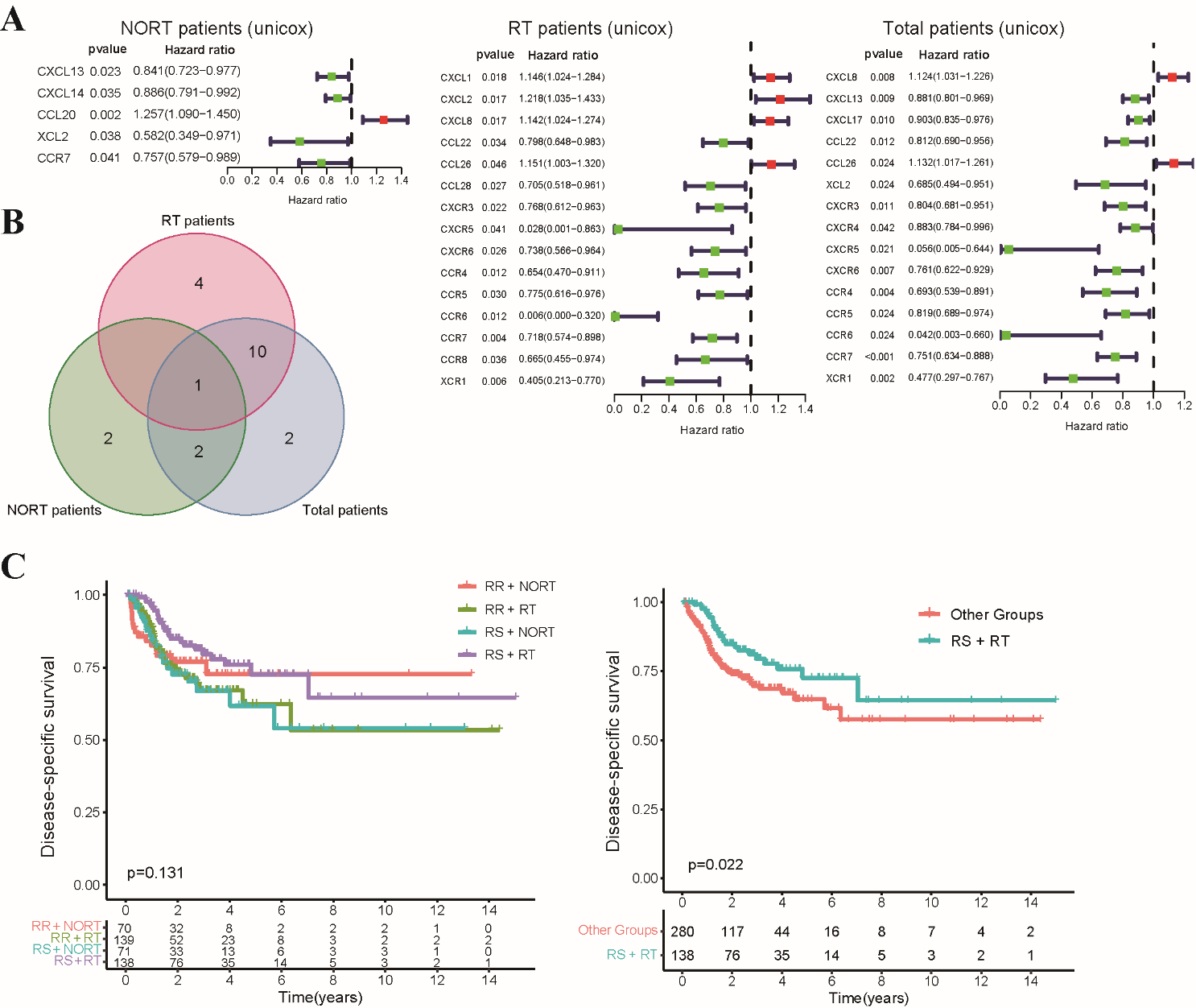


**Figure S2 (A)** Forest plot displaying the results of the univariate Cox regression analysis conducted in the cohort of radiotherapy patients, non-radiotherapy patients and total patients. **(B)** Venn diagram showed that 4 chemokines‑based genes were significantly associated with OS in radiotherapy patients, but not in non-radiotherapy patients and total group. **(C)** The Kaplan-Meier curve shows DSS for RT patients in the RS group compared to all other groups.


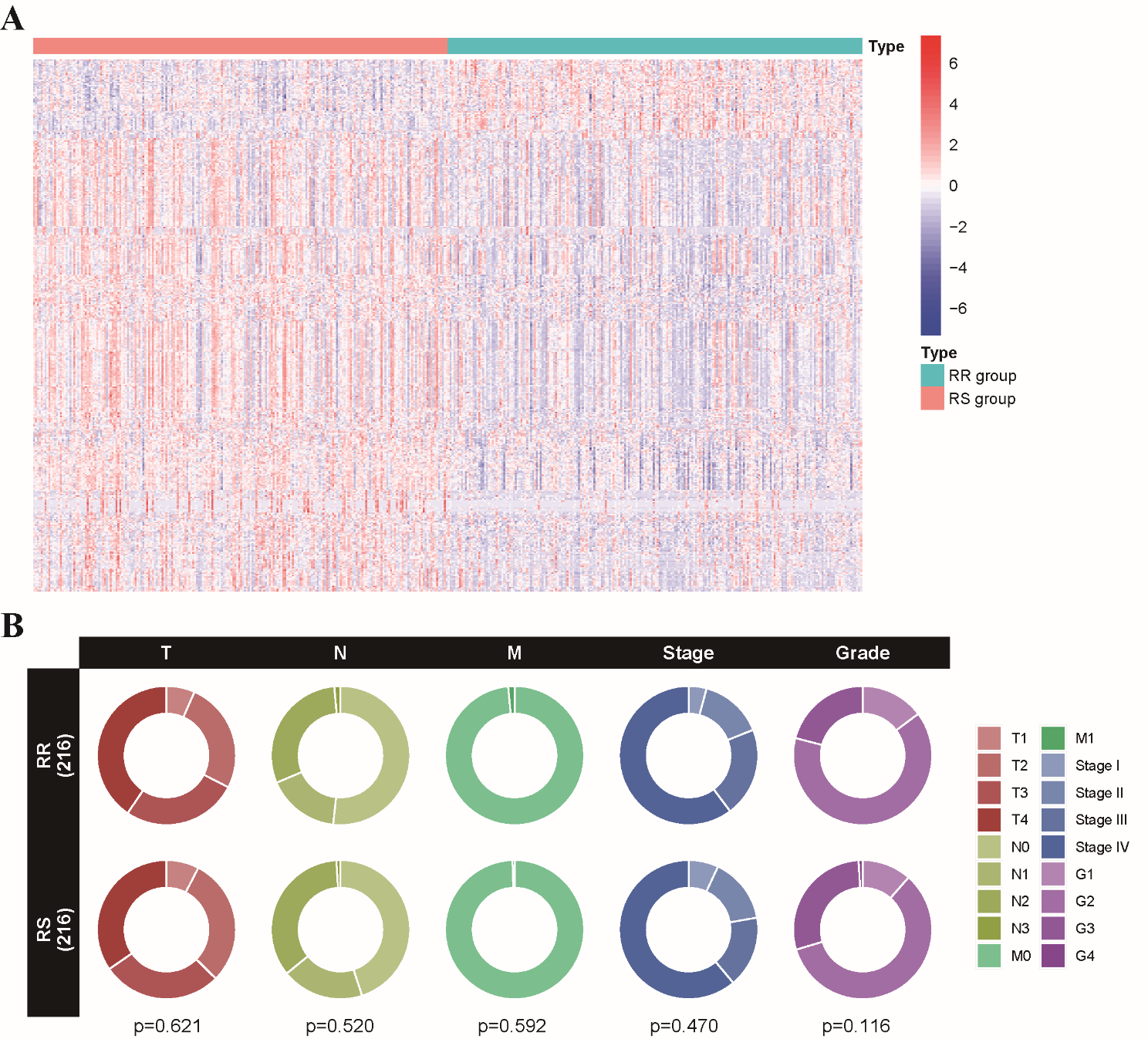


**Figure S3** **(A)** The heatmap displays the differential expression levels of 471 identified DEGs between the RS and RR groups. **(B)** The circus plot depicts the pathological characteristics including T stage, N stage, M stage and pathological grade within the RS and RR groups.

**
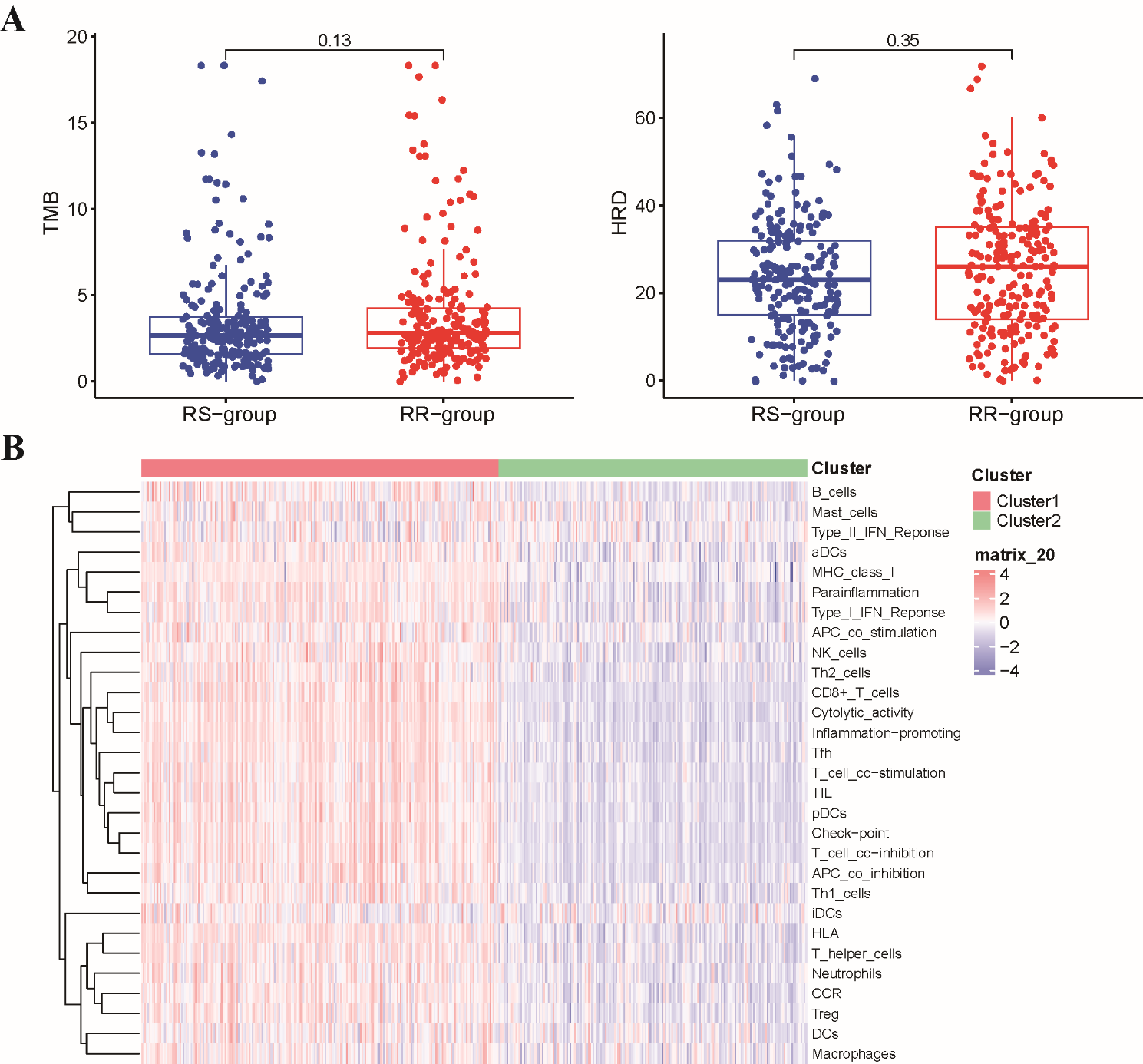
**

**Figure S4** **(A)** The scatter plot shows the distribution of TMB values and HRD scores in the RS and RR groups. **(B)** The heatmap of immune gene sets identified by ssGSEA scores for the two clusters.

**
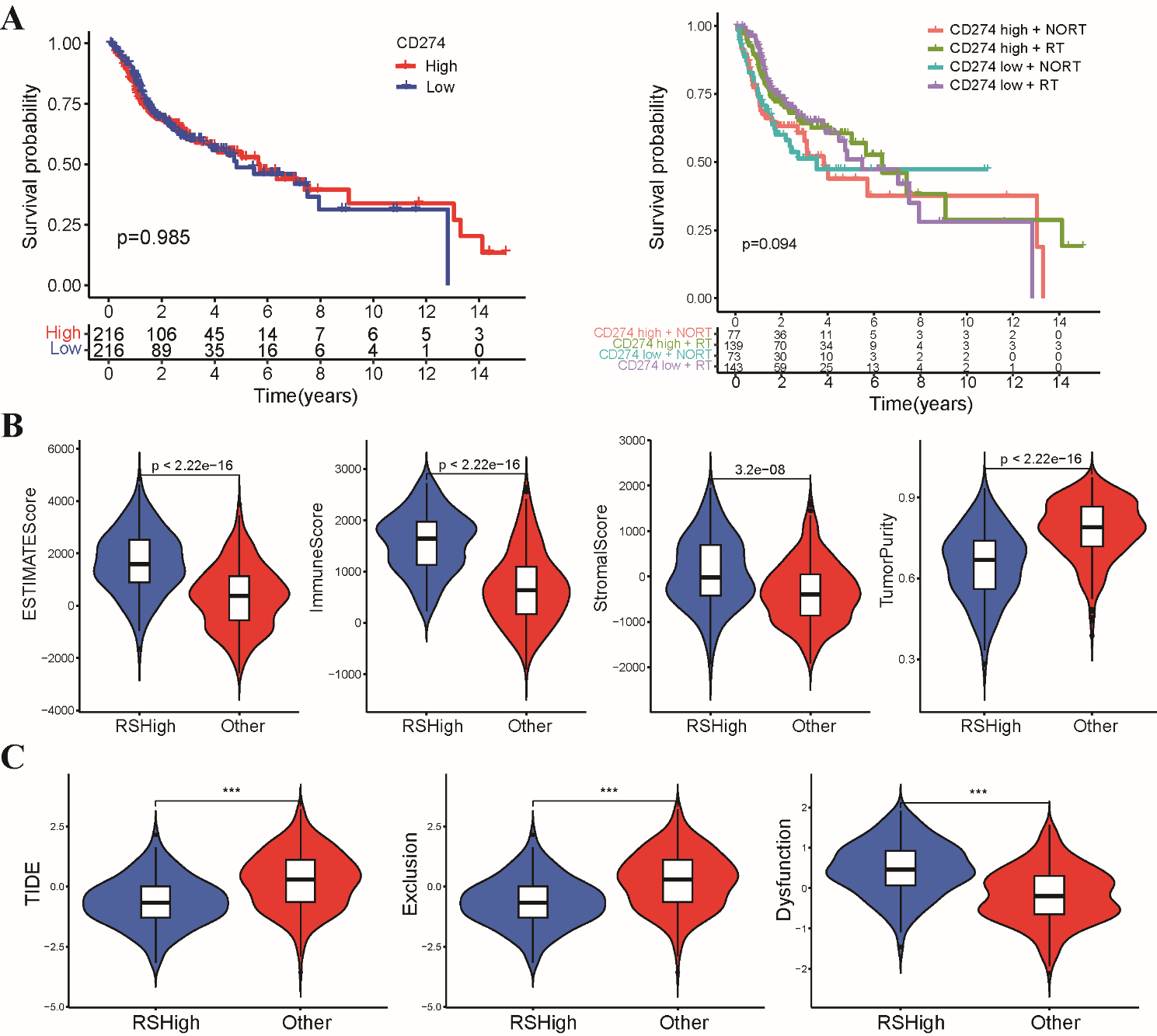
**

**Figure S5** **(A)** Kaplan-Meier curves comparing overall survival rates stratified by the receipt of RT between the PD-L1-high-RR group and the other three patient subgroups. **(B)** The violin plots depict the estimated score, stromal score, immune score, and tumor purity between the PD-L1-high-RR group and the other subgroups. **(C)** The violin plots depict the T cell exclusion, T cell dysfunction, and TIDE scores between the PD-L1-high-RR group and the other subgroups. *** p<0.001.

**Table S1 |** Sequences of the primer used for qRT-PCR

| mRNA |  | Forward primer | Reverse primer |
| --- | --- | --- | --- |
| CXCL2 |  | GGCAGAAAGCTTGTCTCAACCC | CTCCTTCAGGAACAGCCACCAA |
| CCL28 |  | AGCTGTTGCACGGAGGTTTCAC | ACAGCAGCCAAGTCACAATCCC |
| CCR8 |  | TGGCTGTTGTCCATGCCGTGTA | TGGGATGGTAGCCATAATGGCG |
| GAPDH |  | GTCTCCTCTGACTTCAACAGCG | ACCACCCTGTTGCTGTAGCCAA |

The primer sequences used in this study were obtained from OriGene Technologies, Inc.

CCR8 Human qPCR Primer Pair (NM_005201)，CAT#: HP208379

CCL28 Human qPCR Primer Pair (NM_019846)，CAT#: HP213463

CXCL2 Human qPCR Primer Pair (NM_002089)，CAT#: HK202326

GAPDH Human qPCR Primer Pair (NM_002046)，CAT#: HP205798

**Table S2** Association between radiation-related data and radiosensitivity group.

| Covariates | Total cases | RR | RS | Type |
| --- | --- | --- | --- | --- |
| Total doses (Gy) |  |  |  |  |
| <60Gy | 90 (39.3%) | 43 (47.78%) | 47 (52.22%) | Palliative/Adjuvant Radiation |
| ≥60Gy | 139 (60.7%) | 70 (50.36%) | 69 (49.64%) | Curative Radiation |
| Dose per fraction ​ |  |  |  |  |
| 1.8–2.5 Gy/F | 213 (93.0%) | 108(50.70%) | 17 (49.30%) | Conventional Fractionation​ |
| 2.5–5.0 Gy/F | 8 (3.5%) | 2 (25.00%) | 6 (75.00%) | ​​Moderate Hypofractionation |
| ≥5.0 Gy/F | 8 (3.5%) | 3 (37.50%) | 5 (62.50%) | SBRT |
